# Womens’ Work in the Profession

**Published:** 1889-12

**Authors:** Martha J. Rorinson

**Affiliations:** Cleveland, Ohio


### Woman's Work in the Profession.

BY MARTHA J. RORINSON, D.D.S., CLEVELAND, OHIO.

Read before the Ohio State Dental Society, October 30th, 1889.

My subject is so interwoven with the general rights of women that it is almost impossible to discuss a single feature of it without going somewhat over the whole ground of the equality of human rights. It is only within the present century that the sphere of woman has been broadened beyond the boundary of domestic servitude. The prejudice against her has grown out of the fact that in the past, more than to-day, "the might makes right" system has prevailed among all the nations of the world.

It will be assumed, then, that the work of woman in every department of life will be what she can do well, and likes to do. In our own profession she will not be expected to desire and be anxious to extract teeth, but the days of tooth extraction, like the fashion that excluded woman from the arts and various forms of labor in which they are now engaged, are fast passing away. Tooth extraction and all major surgical operations, we relegate to the men, as we do the military and warlike responsibilities, but we do not allow that she is unable to perform all necessary operations upon the teeth because she is unable to endure the privations of camp life or carry a musket.

It is nearly forty years since the first movement was made in Massachusetts in favor of "Woman's Rights," and that may be considered the first step toward the enlarged fields of labor that are now open to her. In every reform the ruling class has always said to those who desired to elevate themselves in society: "You are not fit for the privilege you ask;" and in the dental profession to-day the idea prevails to quite an extent that women are not fit to become dentists, and while they are now admitted to our colleges, that other question is raised—"What work can she best fulfill in her calling?"

That question can best be answered by saying, as I have said before, "Whatever she wants to do, and can do well." Perhaps the care of children's teeth will fall most naturally to her charge. And what greater field for labor is there than that? A much neglected field, too, for so many men think that they have neither the time nor patience to waste with the little folks. The children seem to realize that, and the slight feeling of fear they have on entering the dental office increases as your patience diminishes. No amount of coaxing will make them repeat their visit until, perhaps, that dread toothache comes, and they are forced to come in long enough to have the offender "pulled," not saved.

I think it is true that children will go more naturally to women always, but in my own case I know it to be a fact that I have succeeded in operating for children who would not let several men in the profession even make an examination. Those who have employed women as helpers in dental offices are unanimous in saying that they can get the confidence of exceedingly timid persons better than men; that they have a keener sense of the pain that is necessarily attendant in preparing cavities for filling, and that the delicacy of their touch rather mitigates that inexpressible sensation that is so hard to be endured while using the excavator and engine.

As an assistant she is invaluable, you say; but will she not be equally valuable as a co-worker? Of course she must be properly educated, the same as a man must. A girl who has stood on "the other side" of the chair and merely used a mallet for a few years, would hardly be called a valuable co-worker, but I refer to the educated woman dentist.

It is as difficult to define any rule of labor in dentistry, or any other industry, as it would be to say to the man you *must* do this or that to support yourself and get a living. Every individual should be allowed the largest liberty in selecting his calling, so long as he does not disobey wholesome laws. I was going to say that women are neater than men, but you all, that are present, are such noble exceptions to the rule, that I'll strike it out.

The treating of diseased teeth and gums, and the minor operations belonging to the dental surgeon, the woman is eminently qualified to perform. And in regulating teeth and all prosthetic dentistry, she will not be inferior to man. Women certainly have more patience with women. So if she can save the teeth of those poor nervous sisters who would rather lose them than sit for a couple of hours under a man's hand, she certainly has a place in the profession. But do I hear some one say, "Can she save them?" Of course she can; she can remember that "the vulnerable points in filling an incisor or bicuspid, for instance, are the cervical and lingual borders, just as well as a man can. It is also a fact that woman's eye, from long training, will distinguish shades of color better than most men's will, so she will be less apt to overlook that treacherous white decalcification at those borders.

And she *can* have mechanical ability, and *must* have, to make a success of her work. I know that the majority of women haven't an excess of that necessary quality, because it has not been brought into exercise, but the majority of women would not be dentists if they could. But do give those the opportunity that have the ability. So let me say with Wendell Phillips, "Welcome me henceforth, brother, to your arena; and let facts --not theories--settle my capacity, and therefore, my sphere."

